# Supplementary material for: Pathway-Specific Genomic Alterations in Pancreatic Cancer Across Populations at Risk
Source: Int J Mol Sci. 2025 Aug 8;26(16):7695. doi: 10.3390/ijms26167695 (PMC12386847; doi:10.3390/ijms26167695)
Supplement: Supplementary file 1 [file ijms-26-07695-s001.zip › ijms-3781434-supplementary.pdf]

Supplementary Figures and Tables:

Figure S1. Kaplan-Meier overall survival curves for Non-Hispanic White (NHW) pancreatic cancer patients, stratified by the presence or absence of TGF-Beta (upper left), RTK/RAS (upper middle), WNT (upper right), PI3K (lower left), and TP53 (lower right) pathway alterations.

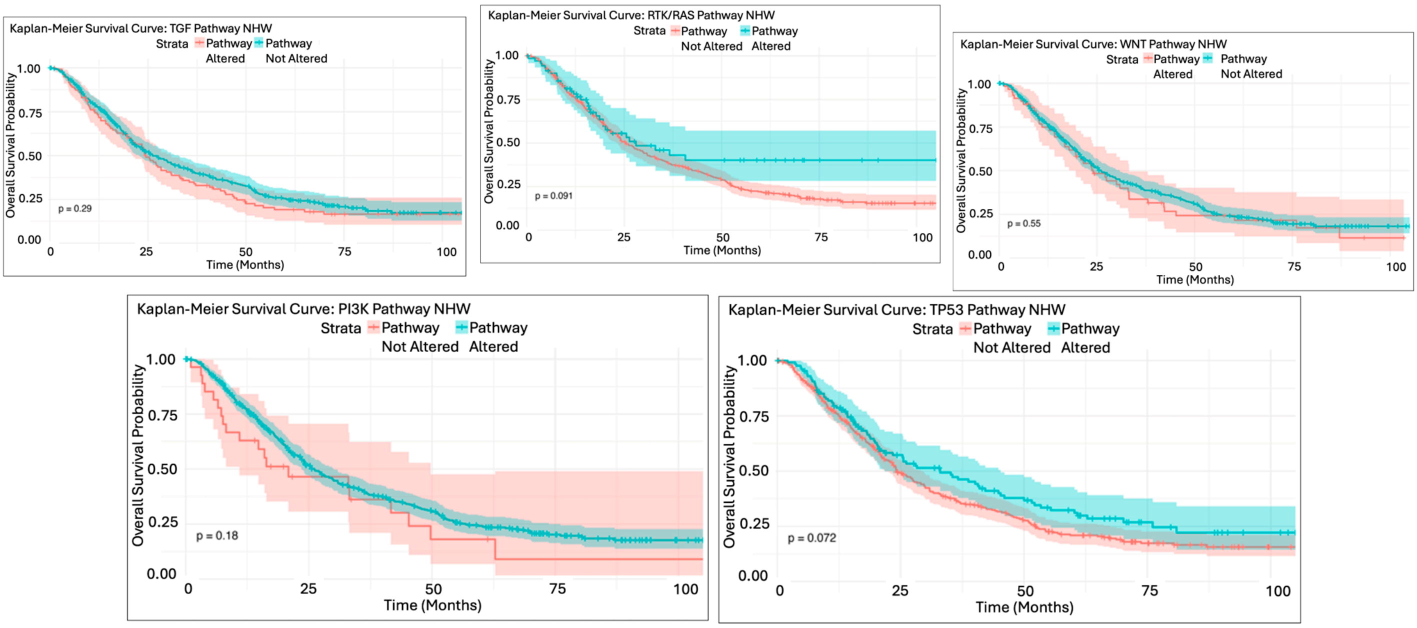

Table S1. Alteration rates of TGF-Beta, RTK/RAS, WNT, PI3K, and TP53 pathway-related genes among Hispanic/Latino (H/L) and Non-Hispanic White (NHW) pancreatic cancer (PC) patients.

TGF-beta Pathway			
**Gene**	**H/L Samples n (%)**	**NHW Samples n (%)**	**p-value**
**ACVR2A Mutation**			
Present	0 (0.0%)	2 (0.1%)	1
Absent	407 (100.0%)	3839 (99.9%)	
**ACVR2B Mutation**			
Present	0 (0.0%)	0 (0.0%)	1
Absent	407 (100.0%)	3841 (100.0%)	
**SMAD2 Mutation**			
Present	6 (1.5%)	14 (0.4%)	**0.006351**
Absent	401 (98.5%)	3827 (99.6%)	
**SMAD3 Mutation**			
Present	2 (0.5%)	50 (1.3%)	0.2317
Absent	405 (99.5%)	3791 (98.7%)	
**SMAD4 Mutation**			
Present	61 (15.0%)	765 (19.9%)	**0.02016**
Absent	346 (85.0%)	3076 (80.1%)	
**TGFBR1 Mutation**			
Present	6 (1.5%)	50 (1.3%)	0.9509

Absent	401 (98.5%)	3791 (98.7%)	
**TGFBR2 Mutation**			
Present	9 (2.2%)	97 (2.5%)	0.8265
Absent	398 (97.8%)	3744 (97.5%)	
**RTK/RAS Pathway**			
**Gene**	**H/L Samples n (%)**	**NHW Samples n (%)**	**p-value**
**EGFR Mutation**			
Present	3 (0.7%)	34 (0.9%)	1
Absent	404 (99.3%)	3807 (99.1%)	
**ERBB2 Mutation**			
Present	4 (1.0%)	49 (1.3%)	0.8146
Absent	403 (99.0%)	3792 (98.7%)	
**ERBB4 Mutation**			
Present	14 (3.4%)	70 (1.8%)	**0.0369**
Absent	393 (96.6%)	3771 (98.2%)	
**MET Mutation**			
Present	8 (2.0%)	34 (0.9%)	0.06705
Absent	399 (98.0%)	3807 (99.1%)	
**PDGFRA Mutation**			
Present	1 (0.2%)	26 (0.7%)	0.5095
Absent	406 (99.8%)	3815 (99.3%)	
**FGFR1 Mutation**			
Present	3 (0.7%)	22 (0.6%)	0.7278
Absent	404 (99.3%)	3819 (99.4%)	
**FGFR2 Mutation**			
Present	3 (0.7%)	21 (0.5%)	0.4964
Absent	404 (99.3%)	3820 (99.5%)	
**FGFR3 Mutation**			
Present	3 (0.7%)	25 (0.7%)	0.7465
Absent	404 (99.3%)	3816 (99.3%)	
**FGFR4 Mutation**			
Present	2 (0.5%)	29 (0.8%)	0.7635
Absent	405 (99.5%)	3812 (99.2%)	
**KIT Mutation**			
Present	7 (1.7%)	31 (0.8%)	0.1134
Absent	400 (98.3%)	3810 (99.2%)	
**IGF1R Mutation**			
Present	3 (0.7%)	28 (0.7%)	1
Absent	404 (99.3%)	3813 (99.3%)	
**RET Mutation**			
Present	6 (1.5%)	50 (1.3%)	0.9509
Absent	401 (98.5%)	3791 (98.7%)	
**ROS1 Mutation**			
Present	5 (1.2%)	44 (1.1%)	1
Absent	402 (98.8%)	3797 (98.9%)	
**ALK Mutation**			
Present	11 (2.7%)	42 (1.1%)	**0.01089**
Absent	396 (97.3%)	3799 (98.9%)	
**FLT3 Mutation**			
Present	6 (1.5%)	30 (0.8%)	0.2435

Absent	401 (98.5%)	3811 (99.2%)	
**NTRK1 Mutation**			
Present	4 (1.0%)	45 (1.2%)	1
Absent	403 (99.0%)	3796 (98.8%)	
**NTRK2 Mutation**			
Present	2 (0.5%)	16 (0.4%)	0.6886
Absent	405 (99.5%)	3825 (99.6%)	
**CBL Mutation**			
Present	1 (0.2%)	33 (0.9%)	0.2495
Absent	406 (99.8%)	3808 (99.1%)	
**ERRFI1 Mutation**			
Present	1 (0.2%)	10 (0.3%)	1
Absent	406 (99.8%)	3831 (99.7%)	
**SOS1 Mutation**			
Present	3 (0.7%)	25 (0.7%)	0.7465
Absent	404 (99.3%)	3816 (99.3%)	
**NF1 Mutation**			
Present	7 (1.7%)	49 (1.3%)	0.6041
Absent	400 (98.3%)	3792 (98.7%)	
**RASA1 Mutation**			
Present	2 (0.5%)	20 (0.5%)	1
Absent	405 (99.5%)	3821 (99.5%)	
**PTPN11 Mutation**			
Present	2 (0.5%)	7 (0.2%)	0.2107
Absent	405 (99.5%)	3834 (99.8%)	
**KRAS Mutation**			
Present	339 (83.3%)	3108 (80.9%)	0.2719
Absent	68 (16.7%)	733 (19.1%)	
**HRAS Mutation**			
Present	5 (1.2%)	5 (0.1%)	**0.000139**
Absent	402 (98.8%)	3836 (99.9%)	
**NRAS Mutation**			
Present	2 (0.5%)	18 (0.5%)	1
Absent	405 (99.5%)	3823 (99.5%)	
**RIT1 Mutation**			
Present	3 (0.7%)	5 (0.1%)	**0.03391**
Absent	404 (99.3%)	3836 (99.9%)	
**ARAF Mutation**			
Present	1 (0.2%)	16 (0.4%)	1
Absent	406 (99.8%)	3825 (99.6%)	
**BRAF Mutation**			
Present	6 (1.5%)	59 (1.5%)	1
Absent	401 (98.5%)	3782 (98.5%)	
**RAF1 Mutation**			
Present	1 (0.2%)	14 (0.4%)	1
Absent	406 (99.8%)	3827 (99.6%)	
**RAC1 Mutation**			
Present	1 (0.2%)	2 (0.1%)	0.2608
Absent	406 (99.8%)	3839 (99.9%)	
**MAPK1 Mutation**			
Present	0 (0.0%)	9 (0.2%)	1

Absent	407 (100.0%)	3832 (99.8%)	
**MAP2K1 Mutation**			
Present	0 (0.0%)	9 (0.2%)	1
Absent	407 (100.0%)	3832 (99.8%)	
**MAP2K2 Mutation**			
Present	3 (0.7%)	15 (0.4%)	0.245
Absent	404 (99.3%)	3826 (99.6%)	
**WNT Pathway**			
**Gene**	**H/L Samples n (%)**	**NHW Samples n (%)**	**p-value**
**AMER1 Mutation**			
Present	4 (1.0%)	29 (0.8%)	0.5516
Absent	403 (99.0%)	3812 (99.2%)	
**APC Mutation**			
Present	12 (2.9%)	91 (2.4%)	0.5803
Absent	395 (97.1%)	3750 (97.6%)	
**AXIN1 Mutation**			
Present	4 (1.0%)	13 (0.3%)	0.07244
Absent	403 (99.0%)	3828 (99.7%)	
**AXIN2 Mutation**			
Present	3 (0.7%)	47 (1.2%)	0.6253
Absent	404 (99.3%)	3794 (98.8%)	
**CTNNB1 Mutation**			
Present	12 (2.9%)	51 (1.3%)	**0.01845**
Absent	395 (97.1%)	3790 (98.7%)	
**DKK1 Mutation**			
Present	0 (0.0%)	0 (0.0%)	1
Absent	407 (100.0%)	3841 (100.0%)	
**DKK2 Mutation**			
Present	0 (0.0%)	0 (0.0%)	1
Absent	407 (100.0%)	3841 (100.0%)	
**DKK3 Mutation**			
Present	0 (0.0%)	0 (0.0%)	1
Absent	407 (100.0%)	3841 (100.0%)	
**DKK4 Mutation**			
Present	0 (0.0%)	1 (0.03%)	1
Absent	407 (100.0%)	3840 (99.97%)	
**GSK3B Mutation**			
Present	0 (0.0%)	8 (0.2%)	1
Absent	407 (100.0%)	3833 (99.8%)	
**LRP5 Mutation**			
Present	0 (0.0%)	1 (0.0%)	1
Absent	407 (100.0%)	3840 (100.0%)	
**LRP6 Mutation**			
Present	0 (0.0%)	2 (0.1%)	1
Absent	407 (100.0%)	3839 (99.9%)	
**RNF43 Mutation**			
Present	16 (3.9%)	209 (5.4%)	0.2392
Absent	391 (96.1%)	3632 (94.6%)	
**SFRP1 Mutation**			
Present	0 (0.0%)	1 (0.0%)	1

Absent	407 (100.0%)	3840 (100.0%)	
**SFRP2 Mutation**			
Present	0 (0.0%)	0 (0.0%)	1
Absent	407 (100.0%)	3841 (100.0%)	
**SFRP3 Mutation**			
Present	0 (0.0%)	0 (0.0%)	1
Absent	407 (100.0%)	3841 (100.0%)	
**SFRP4 Mutation**			
Present	0 (0.0%)	1 (0.0%)	1
Absent	407 (100.0%)	3840 (100.0%)	
**SFRP5 Mutation**			
Present	0 (0.0%)	0 (0.0%)	1
Absent	407 (100.0%)	3841 (100.0%)	
**TCF7 Mutation**			
Present	0 (0.0%)	1 (0.0%)	1
Absent	407 (100.0%)	3840 (100.0%)	
**TCF7L1 Mutation**			
Present	0 (0.0%)	5 (0.1%)	1
Absent	407 (100.0%)	3836 (99.9%)	
**TCF7L2 Mutation**			
Present	1 (0.2%)	11 (0.3%)	1
Absent	406 (99.8%)	3830 (99.7%)	
**TLE1 Mutation**			
Present	0 (0.0%)	1 (0.0%)	1
Absent	407 (100.0%)	3840 (100.0%)	
**TLE2 Mutation**			
Present	0 (0.0%)	2 (0.1%)	1
Absent	407 (100.0%)	3849 (100.2%)	
**TLE3 Mutation**			
Present	0 (0.0%)	3 (0.1%)	1
Absent	407 (100.0%)	3838 (99.9%)	
**TLE4 Mutation**			
Present	0 (0.0%)	1 (0.0%)	1
Absent	407 (100.0%)	3840 (100.0%)	
**WIF1 Mutation**			
Present	0 (0.0%)	1 (0.0%)	1
Absent	407 (100.0%)	3840 (100.0%)	
**PI3K Pathway**			
**Gene**	**H/L Samples n (%)**	**NHW Samples n (%)**	**p-value**
**PTEN Mutation**			
Present	4 (1.0%)	45 (1.2%)	1
Absent	403 (99.0%)	3796 (98.8%)	
**PIK3R1 Mutation**			
Present	2 (0.5%)	30 (0.8%)	0.764
Absent	405 (99.5%)	3811 (99.2%)	
**PIK3R2 Mutation**			
Present	1 (0.2%)	8 (0.2%)	0.5964
Absent	406 (99.8%)	3833 (99.8%)	
**PIK3R3 Mutation**			
Present	1 (0.2%)	3 (0.1%)	0.3317

Absent	406 (99.8%)	3838 (99.9%)	
**PIK3CA Mutation**			
Present	7 (1.7%)	82 (2.1%)	0.7085
Absent	400 (98.3%)	3759 (97.9%)	
**INPP4B Mutation**			
Present	1 (0.2%)	7 (0.2%)	0.5535
Absent	406 (99.8%)	3834 (99.8%)	
**AKT1 Mutation**			
Present	1 (0.2%)	13 (0.3%)	1
Absent	406 (99.8%)	3828 (99.7%)	
**AKT2 Mutation**			
Present	2 (0.5%)	11 (0.3%)	0.3581
Absent	405 (99.5%)	3830 (99.7%)	
**AKT3 Mutation**			
Present	1 (0.2%)	19 (0.5%)	0.7147
Absent	406 (99.8%)	3822 (99.5%)	
**PPP2R1A Mutation**			
Present	1 (0.2%)	14 (0.4%)	1
Absent	406 (99.8%)	3827 (99.6%)	
**TSC1 Mutation**			
Present	3 (0.7%)	25 (0.7%)	0.7465
Absent	404 (99.3%)	3816 (99.3%)	
**TSC2 Mutation**			
Present	10 (2.5%)	75 (2.0%)	0.4566
Absent	397 (97.5%)	3766 (98.0%)	
**STK11 Mutation**			
Present	6 (1.5%)	59 (1.5%)	1
Absent	401 (98.5%)	3782 (98.5%)	
**RHEB Mutation**			
Present	0 (0.0%)	2 (0.1%)	1
Absent	407 (100.0%)	3839 (99.9%)	
**RICTOR Mutation**			
Present	4 (1.0%)	38 (1.0%)	1
Absent	403 (99.0%)	3803 (99.0%)	
**MTOR Mutation**			
Present	8 (2.0%)	43 (1.1%)	0.1466
Absent	399 (98.0%)	3798 (98.9%)	
**RPTOR Mutation**			
Present	4 (1.0%)	28 (0.7%)	0.5419
Absent	403 (99.0%)	3813 (99.3%)	
**TP53 Pathway**			
**Gene**	**H/L Samples n (%)**	**NHW Samples n (%)**	**p-value**
**TP53 Mutation**			
Present	277 (68.1%)	2487 (64.7%)	0.2015
Absent	130 (31.9%)	1354 (35.3%)	
**MDM2 Mutation**			
Present	0 (0.0%)	15 (0.4%)	0.388
Absent	407 (100.0%)	3826 (99.6%)	
**MDM4 Mutation**			
Present	1 (0.2%)	9 (0.2%)	1

Absent	406 (99.8%)	3832 (99.8%)	
**CDKN2A Mutation**			
Present	69 (17.0%)	806 (21.0%)	0.06467
Absent	338 (83.0%)	3035 (79.0%)	
**ATM Mutation**			
Present	8 (2.0%)	151 (3.9%)	0.06441
Absent	399 (98.0%)	3690 (96.1%)	
**CHEK2 Mutation**			
Present	2 (0.5%)	17 (0.4%)	0.7028
Absent	405 (99.5%)	3824 (99.6%)	
**RPS6KA3 Mutation**			
Present	0 (0.0%)	1 (0.0%)	1
Absent	407 (100.0%)	3840 (100.0%)	

Table S2. Nature of gene mutations within the namely TP53, WNT, PI3K, TGF-Beta, and RTK/RAS pathways in pancreatic cancer (PC) among Hispanic/Latino (H/L) and non-Hispanic White (NHW) patients. Mutation types include frame shift deletions, frame shift insertions, missense mutations, nonsense mutations, splice site mutations, and translation start site mutations.

	HL Samples							
	Frame Shift Deletion	Frame Shift Insertion	In Frame Deletion	In Frame Insertion	Missense Mutation	Nonsense Mutation	Splice Region	Splice Site
AXIN1	0.0%	0.0%	0.0%	0.0%	75.0%	0.0%	0.0%	25.0%
HRAS	0.0%	0.0%	0.0%	0.0%	100.0%	0.0%	0.0%	0.0%
SMAD2	0.0%	0.0%	0.0%	0.0%	66.7%	16.7%	0.0%	16.7%
SMAD4	16.7%	10.0%	1.7%	0.0%	35.0%	33.3%	1.7%	1.7%
CTNNB1	0.0%	0.0%	0.0%	0.0%	91.7%	8.3%	0.0%	0.0%
ERBB4	0.0%	0.0%	0.0%	0.0%	76.9%	7.7%	7.7%	7.7%
ATM	0.0%	0.0%	0.0%	0.0%	75.0%	8.3%	8.3%	8.3%
ALK	0.0%	0.0%	0.0%	0.0%	100.0%	0.0%	0.0%	0.0%
CDKN2A	19.7%	7.0%	4.2%	1.4%	31.0%	33.8%	0.0%	2.8%
MET	0.0%	0.0%	0.0%	0.0%	100.0%	0.0%	0.0%	0.0%
RIT1	0.0%	0.0%	0.0%	0.0%	100.0%	0.0%	0.0%	0.0%

	NHW Samples									
	Frame Shift Deletion	Frame Shift Insertion	In Frame Deletion	In Frame Insertion	Missense Mutation	Nonsense Mutation	Nonstop Mutation	Splice Region	Splice Site	Translation Start Site
AXIN1	28.6%	0.0%	0.0%	0.0%	35.7%	28.6%	0.0%	0.0%	7.1%	0.0%
HRAS	0.0%	0.0%	0.0%	0.0%	80.0%	0.0%	0.0%	20.0%	0.0%	0.0%
SMAD2	0.0%	7.1%	0.0%	0.0%	78.6%	14.3%	0.0%	0.0%	0.0%	0.0%
SMAD4	15.1%	11.6%	2.0%	0.4%	39.9%	25.9%	0.5%	0.6%	3.9%	0.0%
CTNNB1	0.0%	0.0%	1.8%	0.0%	93.0%	0.0%	0.0%	5.3%	0.0%	0.0%
ERBB4	0.0%	0.0%	0.0%	0.0%	84.0%	8.0%	0.0%	5.3%	2.7%	0.0%
ATM	11.3%	6.8%	0.6%	0.0%	48.6%	17.5%	0.0%	4.0%	10.7%	0.6%
ALK	2.3%	0.0%	2.3%	0.0%	83.7%	2.3%	0.0%	0.0%	9.3%	0.0%
CDKN2A	27.1%	11.0%	5.9%	1.3%	26.5%	22.5%	0.0%	0.2%	5.4%	0.1%
MET	0.0%	0.0%	0.0%	0.0%	100.0%	0.0%	0.0%	0.0%	0.0%	0.0%
RIT1	0.0%	0.0%	0.0%	0.0%	60.0%	0.0%	0.0%	40.0%	0.0%	0.0%

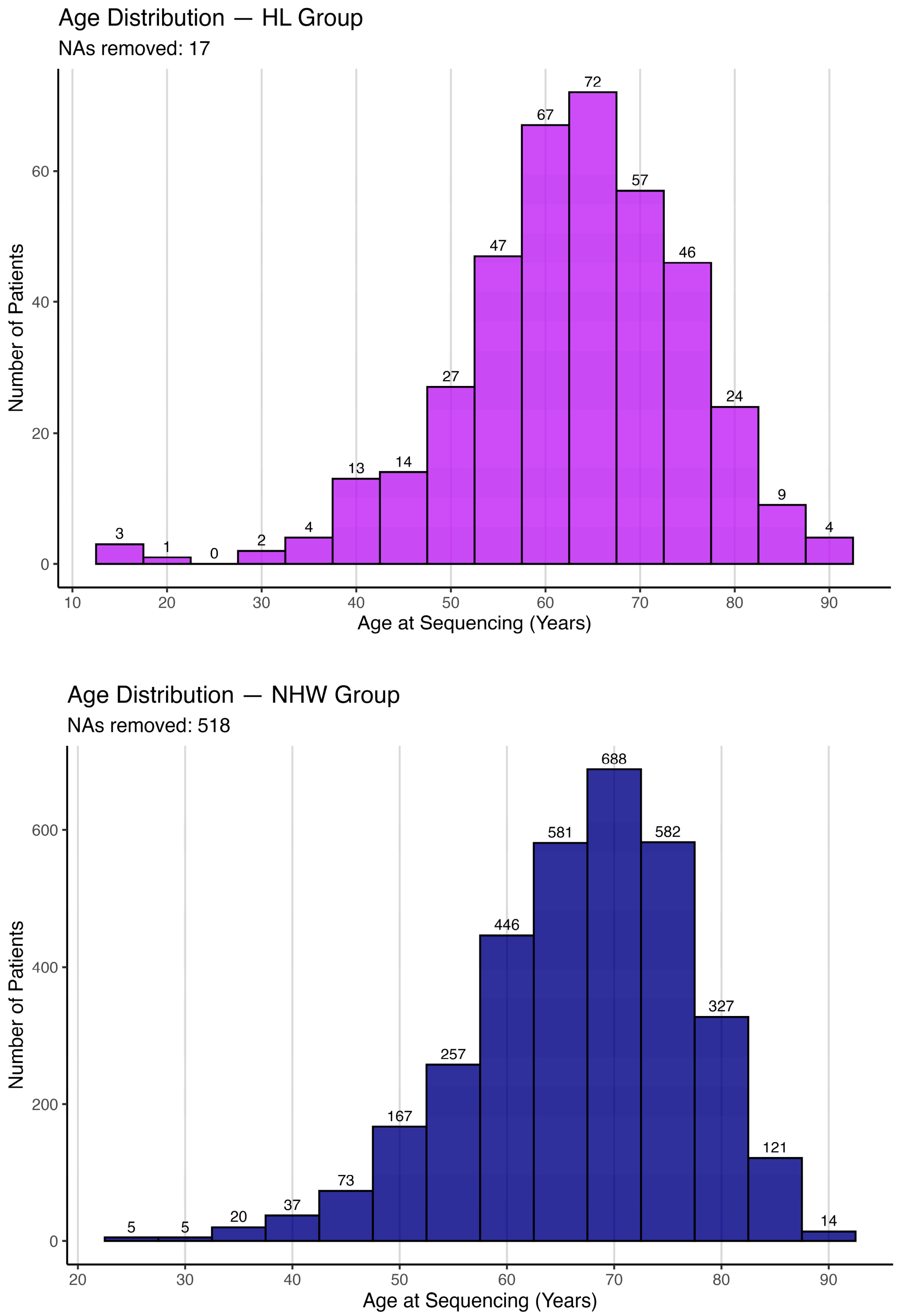

Figure S2. Age Distribution of Pancreatic Cancer Patients by Ethnic Group. Histograms showing the age at sequencing for pancreatic cancer patients in the (top) Hispanic/Latino (H/L) group and (bottom) Non-Hispanic White (NHW) group. The H/L cohort (N = 407) displays a broader age distribution with a peak around 65 years and a notable proportion of patients diagnosed before age 50. In contrast, the NHW cohort (N = 3,841) exhibits a unimodal distribution skewed older, peaking at 70 years. Missing age data were excluded (17 H/L; 518 NHW).
